# Clinical characteristics and outcomes of gastrointestinal stromal tumor patients receiving surgery with or without TKI therapy: a retrospective real-world study

**DOI:** 10.1186/s12957-023-02897-y

**Published:** 2023-01-23

**Authors:** Lingquan Wang, Zhentian Ni, Wei Xu, Yu Mei, Chen Li, Zhenggang Zhu, Wentao Liu

**Affiliations:** 1 Department of General Surgery, Shanghai Institute of Digestive Surgery, Ruijin Hospital, Shanghai Jiao Tong University School of Medicine, Shanghai, 200025 China; 2 Shanghai Key Laboratory of Gastric Neoplasms, Shanghai Jiao Tong University School of Medicine, Shanghai, 200025 China

**Keywords:** Gastrointestinal stromal tumor (GIST), Surgical treatment, TKI-targeted therapy, Prognostic factors, Ki-67

## Abstract

**Purpose:**

To retrospectively analyze the clinical characteristics of patients undergoing surgical treatment for gastrointestinal stromal tumors (GISTs) in Ruijin Hospital and explore the relevant prognosis clinical factors after surgical treatment.

**Methods:**

We screened out 1015 patients with GISTs diagnosed and treated during January 2010 to December 2019. We performed univariate analysis by the log-rank test and multivariate analysis by COX regression. The Kaplan–Meier method was used to estimate the disease-free survival (DFS) and overall survival (OS) of the whole group.

**Results:**

All 1015 patients in the whole group received radical surgery, and the proportion of patients with high, intermediate, and low risk was 31.1%, 21.7%, and 47.3%, respectively. Among the 480 low-risk patients, surgery could achieve radical therapy; only the Ki-67 index was related to DFS and OS (DFS: *p* = 0.032, OS: *p* = 0.009) among the 140 intermediate-risk patients with tumors located in the stomach, whether received Tyrosine kinase inhibitors (TKIs) therapy did not affect the prognosis of patients (DFS: *p* = 0.716, OS: *p* = 0.848). Among the 331 high-risk patients, those with non-gastric tumors (those outside the stomach, duodenum, and small intestine, HR 1.55, 95% CI 1.19–2.00, *p* < 0.001), tumor diameter > 10 cm (hazard ratio, HR 2.63, 95% confidence interval, CI 2.09–4.03, *p* < 0.001), as well as high-risk patients with mitotic rate > 10/50 HPF (HR 2.74, 95% CI 2.00–3.76, *p* < 0.001), the overall prognosis was obviously worse than that of other patients. For some high-risk patients, prolonged postoperative imatinib therapy could significantly improve the survival of patients (HR 0.43, 95% CI 0.15–0.66, *p* < 0.001).

**Conclusions:**

For the vast majority of GIST patients, surgery can be curative; but in intermediate-risk patients, the Ki-67 index and postoperative TKI treatment are closely related to prognosis. For intermediate-risk patients whose primary tumor is the stomach, the value of TKI-targeted therapy after surgery seem be not necessary in our study. However, for some high-risk patients, the prognosis of patients can be improved by appropriately prolonging the treatment time of TKI.

**Supplementary Information:**

The online version contains supplementary material available at 10.1186/s12957-023-02897-y.

## Introduction

Gastrointestinal stromal tumors (GISTs) are the most common mesenchymal tumors of the gastrointestinal tract, though they account for less than 5% of gastrointestinal malignancies [1]. GISTs arise primarily from interstitial cells of Cajal (ICC) and have been shown to be primarily caused by activating mutations in *KIT* or *PDGFRA* tyrosine kinase receptors(TKIs) in nearly 85–90% of patients [2, 3]. As GISTs are potentially malignant tumors, recurrence and metastasis are possible, but the prognosis differs among patients with different tumor locations; the prognosis is mainly affected by the tumor diameter and mitotic image [4, 5].

For GIST patients, surgical resection is currently the first choice for radical resection [6]. Considering that there is almost no lymph node metastasis in GISTs [7], the surgical approach is different from conventional radical resection of malignant tumors. The current surgical methods mainly include: traditional open surgery, minimally invasive surgery (laparoscopic surgery, da Vinci surgery), endoscopic resection, and double-endoscopy combined surgery (digestive endoscopy + laparoscopy). With the widespread popularity of minimally invasive surgery, for GIST surgery, a number of clinical studies from many centers [8–10] have shown that minimally invasive surgery is not only safe but also produces no significant difference in patient survival compared with traditional open surgery. Therefore, it is recommended that in the case of radical surgery, the chief surgeon should choose a less invasive surgical method if possible.

Although radical surgery can completely cure a GIST, only 60–70% of patients can fully benefit from surgery; 30–40% of patients experience recurrence or metastasis after surgery [11, 12], and GIST cannot be benefit from conventional chemoradiotherapy. In 2002, the Food and Drug Administration (FDA) approved imatinib (Imatinib, IM, a tyrosine kinase inhibitor) for the targeted therapy of unresectable/metastatic GISTs. Since then, the treatment mode of GIST has officially entered the imatinib era, and the overall prognosis of patients has improved significantly [13]. The median survival time has increased from 19 to 57 months [14]. For postoperative adjuvant therapy, previous randomized controlled studies [15, 16] have shown that in intermediate- and high-risk patients, 1-year postoperative imatinib treatment only affects postoperative disease-free survival (DFS) time but not overall survival (OS) time. However, for high-risk patients, Imatinib-targeted therapy after primary tumor surgery can significantly prolong surgical RFS and OS for 3 years [17]. Currently, the standard postoperative treatment plan recommended in China is targeted therapy for no less than 1 year after surgery for intermediate-risk patients and targeted therapy for no less than 3 years after surgery for high-risk patients [18].

To study the relevant factors related with the prognosis of patients after surgery, we performed the research to study the clinical features of GIST patients under real-world conditions in Ruijin Hospital from 2010 to 2019.

## Methods

### Patients and tumor samples

By retrieving the pathology and medical history system of our hospital, we screened out all patients with primary GISTs diagnosed and treated from January 2010 to December 2019, and other tumors were excluded. The specific inclusion criteria of this study were as follows: (1) postoperative pathological immunohistochemistry confirmed the surgical specimen as positive for CD117 (KIT) and/or DOG-1; (2) GIST patients were under 80 years old at the time of initial diagnosis; (3) no patients had received any treatment before treatment in our hospital, and accepted to be treated in the hospital; and (4) patients had complete clinical and pathological data and could accurately judge the degree of risk. The exclusion criteria were as follows: (1) patients who underwent surgery had recurrent or metastatic tumors; (2) patients who underwent neoadjuvant therapy could not be evaluated for tumor risk after surgery; (3) patients who died of perioperative complications within 30 days after surgery; and (4) patients who only received puncture diagnosis without surgical resection.

In addition, the surgical status (surgical method, resection margin, surgery-related indicators: time, intraoperative blood loss, postoperative complication rate [19], etc.), postoperative pathology (risk level, immunohistochemical indicators), effective follow-up time (operation time, disease recurrence, or metastasis time, death time, final follow-up time), and mode of Imatinib therapy were also collected completely. The mitotic image [20] was judged as the number of mitotic images per 50 high-power microscopes by the pathologist. Currently, the most common postoperative risk assessment system in the clinic is based on the 2017 revised Chinese consensus of NIH 2008 [21]. Therefore, we classified the primary GIST by different tumor locations, tumor diameters, and mitotic images to classify the GIST risk. We divided the GIST risk into the following categories: very low, low, intermediate, and high. This retrospective study was approved by the Ethics Committee of Ruijin Hospital, affiliated with Shanghai Jiao Tong University School of Medicine; all enrolled patients signed the relevant informed consent.

### Data collection and follow-up

**Fig. 1:**
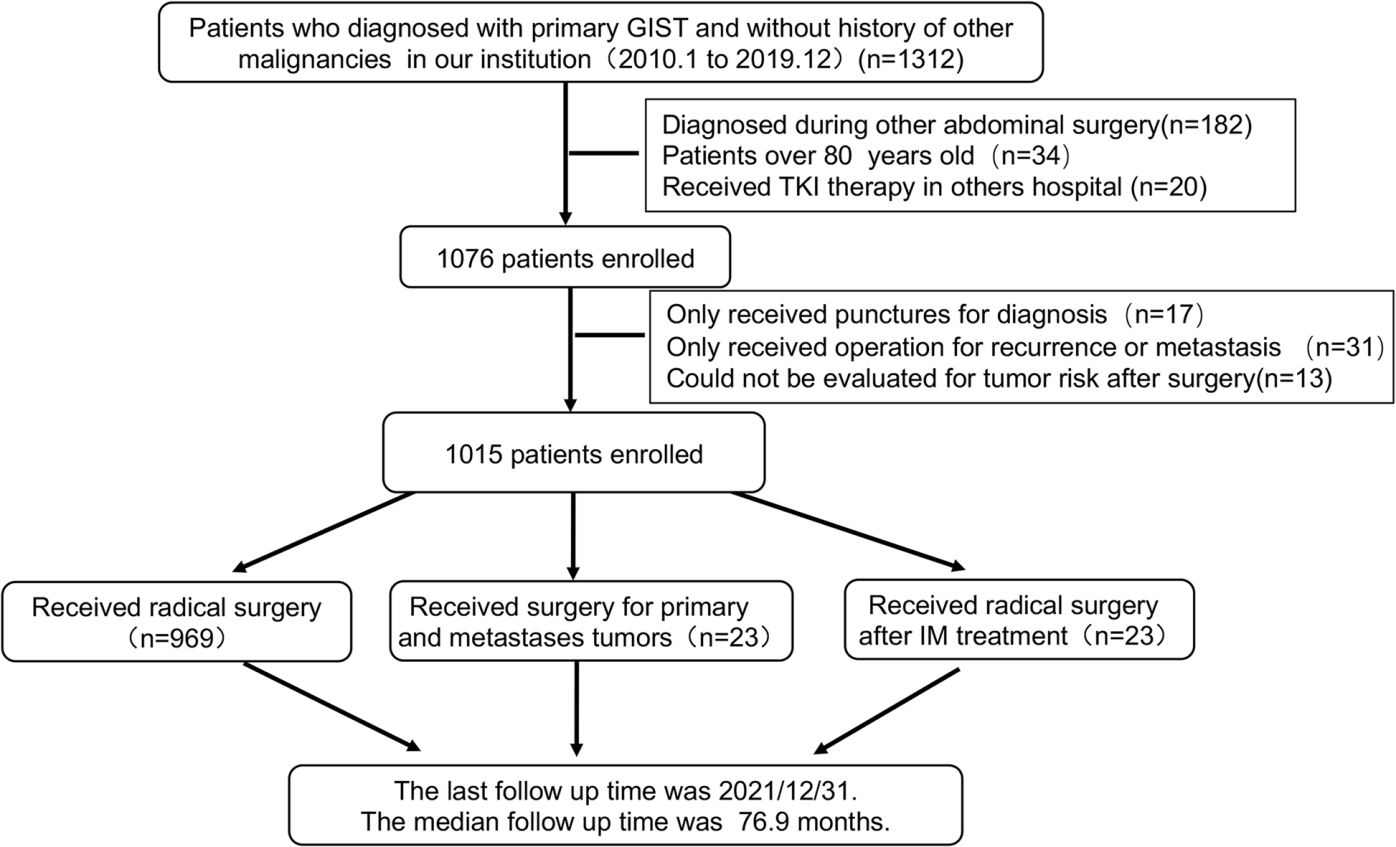
Flowchart of patient selection process

From January 1, 2010, to December 31, 2019, a total of 1312 GIST patients were treated in our hospital. After screening for inclusion criteria, 297 patients were excluded because they did not meet the inclusion criteria, and a total of 1015 GIST patients were finally included in the study (Fig. 1). The last follow-up date for this study was December 31, 2021.

The primary method of follow-up in this retrospective study was outpatient and telephone long-term follow-up of postoperative patients. The outpatient follow-up mainly focused on the patients’ subjective symptoms, physical examination, routine blood test items (blood routine, liver and kidney function, electrolytes, DIC), chest and abdomen plain scan + enhanced CT (computer tomography), and gastrointestinal endoscopy. Moreover, if the condition required MRI (magnetic resonance imaging), a whole-body bone scan and whole-body PET-CT (positron emission computed tomography) examination were added. For intermediate- and high-risk patients, outpatient or outpatient follow-up was conducted every 3 months during the first 3 years after surgery, and after 3 years after surgery, telephone or outpatient follow-up was conducted on average every 6 months. In order to investigate the safety of different surgery methods among low-risk patients, we taken the operation time, bleeding loss volume, rate of convert to another method, rate of postoperative complications, recovery feeding time, rate of re-reoperation and hospital stay time into consideration. To explore the relevant factors affecting the prognosis of patients, we performed univariate and multivariate analyses on clinical indicators; including gender, age, BMI, presence or absence of anemia, tumor location, tumor diameter, risk degree, histological type, Ki-67, surgical methods, and TKI treatment. The main contents of the follow-up included the presence or absence of tumor recurrence as well as survival. According to the follow-up, the tumor recurrence time and death time of each group of patients were determined. The primary endpoint of this study mainly included the OS time of the patients, and the secondary endpoint was the DFS time. In contrast, we defined OS as the time from the date a patient was diagnosed with GIST (including surgery or biopsy) to the date of death or the last follow-up. DFS was defined as the time from the date of radical or extended radical surgery in a GIST patient to the date of disease recurrence, date of progression, or last follow-up.

### Statistical analysis

Statistical data were divided into technical data and measurement data in our research. For count data, the frequency was used; for measurement data, if they conformed to a normal distribution, they were expressed as the mean ± standard deviation. They were expressed as the median (minimum to maximum). In univariate analysis, for enumeration data, we used Fisher's exact test to analyze the differences between patients in different groups; for quantitative data, if the clinical data conformed to a normal distribution, an independent sample *t* test was used to analyze the differences between patients in different groups. If the differences were not in line with the normal distribution, the non-parametric test was used to analyze the differences between patients in different groups. For the patients, OS and DFS were calculated by the Kaplan–Meier (KM) method, and the log-rank test was used to test whether there was a statistical difference. After multivariate analysis identified statistical differences and calculated hazard ratios (HRs), they were determined to be independent prognostic factors. We used SPSS software version 27.0 (IBM Statistical Product and Service Solutions, Armonk, USA, 2021) for statistical analysis of the data; *p* < 0.05 was considered as a statistically significant difference. In addition, we used GraphPad 9.0 software to draw the KM survival curves of patients in different groups.

## Results

### Research population and clinical features

We found that in the whole group of 1015 patients, the male-to-female ratio was 1.08:1, the median age at diagnosis was 58.79 years, and the median preoperative BMI was 22.78 (13.96–33.68). The highest proportion of patients had a primary tumor in the stomach (59.3%), followed by the small intestine (24.3%), duodenum (9.1%), colorectum (5.0%), retroperitoneum (1.4%), and esophagus (0.9%). In the histological type, 78.1% of the tumors were the spindle-cell type, while epithelioid and mixed types represented 10% and 9.7% of tumors, respectively. Because some patients received imatinib before surgery, in 22 cases (2.2%), postoperative pathological specimens could not determine the histological type. In the whole group of patients, 101 patients (10%) had anemia before surgery, including 45 patients (4.4%) with moderate to severe anemia. The proportions of patients with high, intermediate, and low risk were 32.6%, 20.1%, and 47.3%, respectively. The surgical specimens of all patients were examined by immunohistochemistry (including CD117, Dog-1, CD34, and Ki-67).

Among the patients in the whole group received surgical treatment, 572 patients underwent local tumor resection (including 53 patients who underwent endoscopic resection, ESR), 311 patients underwent tumor resection combined with gastrointestinal reconstruction, and 132 patients had a tumor invading surrounding organs or single metastasis. Some patients underwent extended radical mastectomy (including 23 patients who underwent primary tumor + single metastases resection). In the whole group of patients, 530 patients did not receive TKI treatment after surgery, including 480 low-risk patients, 41 intermediate-risk patients, and 9 high-risk patients, of which intermediate- and high-risk patients did not receive TKI therapy. The reasons for taking the drug included age, liver and kidney insufficiency, and personal reasons. Moreover, 341 patients received regular TKI therapy, including 64 patients who had targeted therapy for more than 3 years). In addition, 73 patients did not receive regular TKI treatment, of which 56 patients stopped after drug side effects (e.g., moderate and severe bone marrow suppression, liver and kidney function damage, moderate and severe soft tissue edema, heart failure). The remaining patients stopped taking the drug for financial reasons.

### Prognosis factors among different risk-degree patients

Among the whole group, the survival analysis shown in supplement file-1, and analysis of clinical features and related factors among low-risk patients with different surgical methods were presented in supplement file-1.

**Fig. 2:**
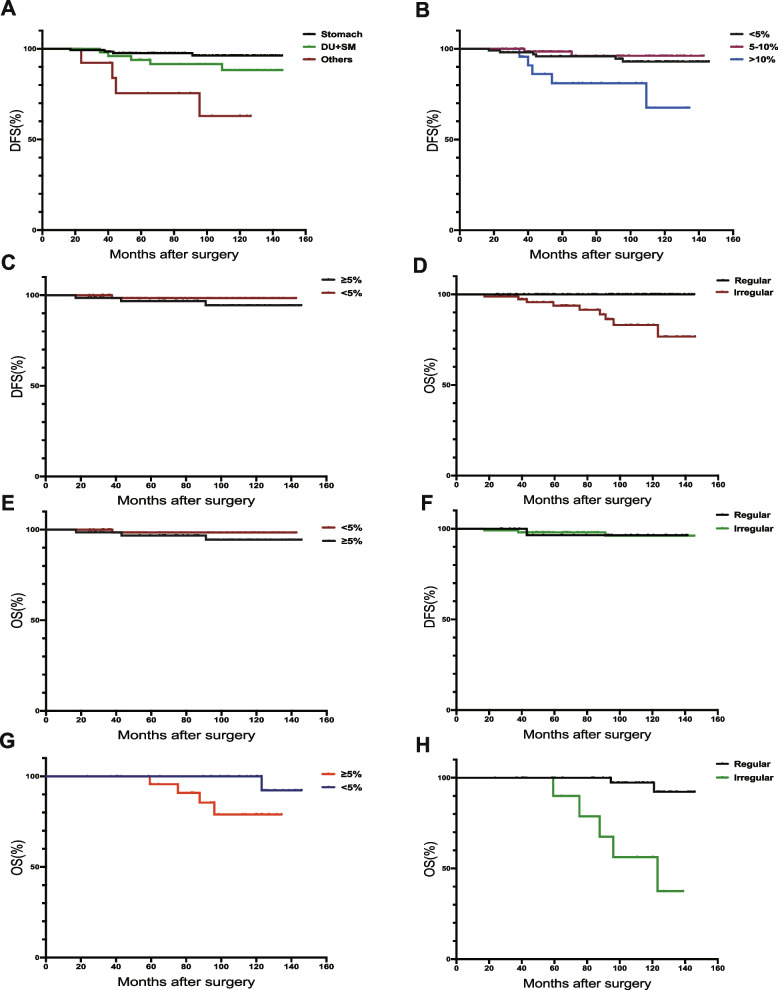
Overall and disease-free survival analysis of 204 patients diagnosed with middle risk: disease-free survival of patients with different sites in the whole group of patients (**A**); disease-free survival of different Ki-67 indices (**B**); disease-free survival of 140 patients with tumors located in the stomach with different Ki-67 indices (**C**); the overall survival of the whole group of patients with different TKI treatment methods (**D**); the overall survival of 140 patients with tumors located in the stomach with different Ki-67 indices (**E**) the overall survival of 140 patients with tumors located in the stomach with different TKI treatment methods (**F**); the overall survival of 64 patients with different Ki-67 indices in non-stomach tumors (**G**); the overall survival of 64 patients with tumors located in non-stomach with different IM treatment methods (**H**)

In addition, for patients requiring TKI treatment (intermediate and high risk in the modified NIH version), we also found that TKI therapy was also an independent factor affecting postoperative recurrence. Among these 535 patients, 204 were at intermediate risk and 331 were at high risk (see Table 1 for details). In addition to significant differences in clinical factors (tumor diameter, mitotic figures, tumor location), we also found statistically significant differences in gender, histological classification, and TKI treatment. For the prognostic factors among intermediate- and high-risk patients, we found that only the tumor location (*p* < 0.001, Fig. 2A) and Ki-67 index (*p* = 0.003, Fig. 2B) were associated with postoperative DFS, but only TKI treatment was associated with OS (*p* < 0.001, Fig. 2D). Two hundred four intermediate-risk patients were divided into gastric group (Stom group) and non-gastric group (non-Stom group), we found significant differences between the two groups in gender, histological classification, tumor diameter, mitotic figures, Ki-67 index, and TKI treatment patterns.

**Table 1:** Clinical characteristics of 204 intermediate- and 331 high-risk GIST patients

Characteristic		Middle (*n* = 204)	High(*n* = 331)	*P* value
Age	≤ 60	102	174	0.565
	> 60	102	157	
Sex	Male	90	196	0.001
	Female	114	135	
BMI (kg/m^2^)	≤ 22.78	101	139	0.908
	> 22.78	103	19	
Anemia	Yes	22	34	0.401
	No	182	277	
Primary tumor site	Stomach	140	141	< 0.001
	DU + SM	51	154	
	Others	13	36	
Tumor size(cm)	< 5	70	47	< 0.001
	5–10	133	176	
	> 10	1	108	
Histological variant	Spindle	162	207	0.032
	Epithelioid	25	71	
	Mixed	17	53	
Mitotic rate	< 5	134	94	< 0.001
	5–10	68	152	
	> 10	2	85	
Ki-67 index(%)	< 5	104	80	< 0.001
	5–10	77	89	
	> 10	23	152	
TKI therapy	Regular	133	281	< 0.001
	Irregular	30	41	
	No drug	41	9

**Fig. 3:**
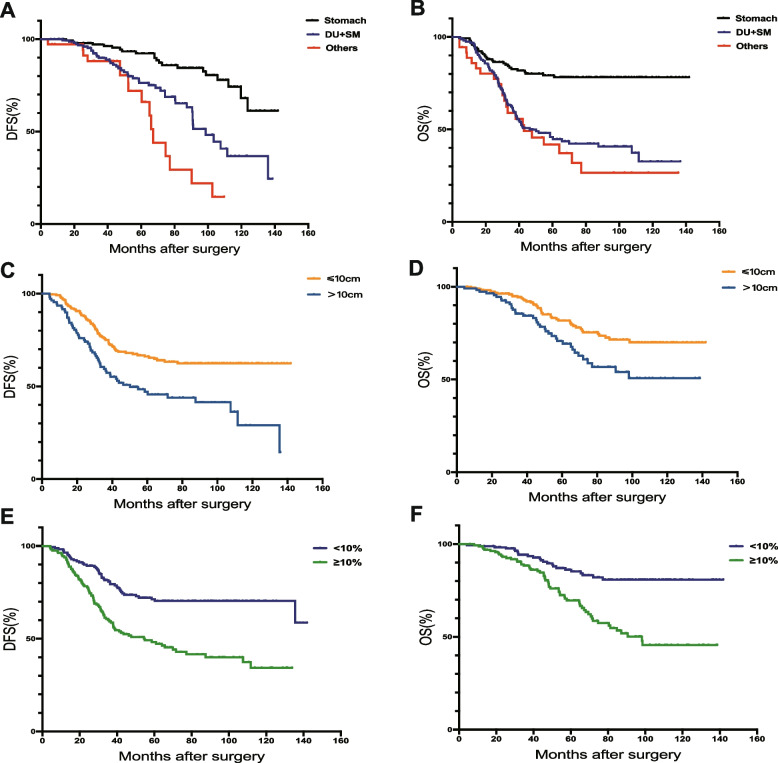
Overall and disease-free survival analysis of 331 patients diagnosed with high risk: disease-free survival (**A**) and overall survival (**B**) of patients in different site of the 331 patients; disease-free survival (**C**) and overall survival (**D**) of patients with different tumor diameters in each group; different Ki-67 index of the disease-free survival (**E**) and overall survival (**F**) of each group

As for different tumor locations could influenced the prognosis of intermediate-risk patients. So, we analyzed intermediate-risk patients with different tumor locations separately. However, in 140 intermediate-risk patients with tumors located in the stomach, we found that only the Ki-67 index was associated with postoperative DFS. It was related to OS (DFS: *p* = 0.032, Fig. 2C; OS: *p* = 0.009, Fig. 2E), but interestingly, the presence or absence of TKI treatment did not affect patients’ overall prognosis (DFS: *p* = 0.716, OS: *p* = 0.848, Fig. 2F). For patients whose primary tumor site was non-gastric, DFS-related factors included the Ki-67 index (*p* < 0.001) and surgical approach (*p* = 0.008), but TKI treatment (*p* < 0.001) and the Ki-67 index (*p* < 0.001) were closely related to OS (Fig. 2G, H). Of the 331 patients in the high-risk group, 281 received regular perioperative TKI therapy; of the remaining 50 high-risk patients, 41 did not complete regular targeted therapy, and 9 patients did not receive postoperative adjuvant therapy. Then, we performed univariate prognostic analysis on 331 high-risk patients (Table 2), and we found that there was no statistical difference between gender, age, and the presence or absence of anemia and the prognosis of patients. In contrast, the tumor location, tumor diameter, Ki-67 index, mitotic image, and TKI treatment methods were closely related to prognosis. Additionally, different surgical methods were only related to the OS time of patients and had little effect on the DFS of patients. For the whole group of 331 high-risk patients, the survival curve is shown in Fig. 3. We found that patients with tumors located in non-stomach (small intestine, duodenum, colorectum, and others) had significantly worse prognosis than those located in the stomach (Fig. 3A, B), patients with tumor diameter > 10 cm had significantly worse prognosis than those with ≤ 10 cm (Fig. 3C, D); as for Ki-67 index, ≤ 10% of patients had a better prognosis than patients with index > 10% (Fig. 3E, F).

**Table 2:** Univariate survival analysis of 331 high-risk GIST patients

Variable	DFS		OS	
	HR (95%CI)	*P* value	HR (95%CI)	*P* value
Age				
≤ 60	1		1	
60	1.25(0.88–1.53)	0.147	1.19(0.84–1.71)	0.212
Sex				
Male	1		1	
Female	1.05(0.79–1.19)	0.412	0.93(0.56–1.27)	0.670
BMI (kg/m^2^)				
22.78	1		1	
≤ 22.78	0.96(0.67–1.37)	0.198	1.17(0.91–1.43)	0.219
Tumor size(cm)				
≤ 10	1		1	
10	4.04(2.67–6.79)	< 0.001	3.74(2.42–5.43)	0.003
Tumor site				
Stomach	1		1	
DU + SM	1.71(1.45–2.43)	0.009	1.48(1.16–1.89)	0.017
Others	2.49(1.35–4.34)	< 0.001	1.77(1.59–2.18)	< 0.001
Histological variant				
Spindle	1		1	
Epithelioid	0.87(0.74–1.40)	0.122	0.69(0.54–1.27)	0.208
Mixed	1.08(1.01–1.33)	0.028	0.97(0.95–1.09)	0.091
Surgery				
Local excision	1		1	
Contained DTE	1.26(0.89–1.73)	0.011	1.11(1.09–1.24)	0.032
Extensive radical surgery	1.86(0.91–2.42)	< 0.001	2.47(1.71–3.39)	< 0.001
Ki-67 index(%)				
≤ 10	1		1	
10	3.14(2.40–4.67)	< 0.001	2.43(1.96–3.51)	< 0.001
Mitotic rate				
≤ 10/50	1		1	
10/50	2.79(2.16–4.08)	< 0.001	2.13(1.76–3.09)	< 0.001
TKI therapy				
Regular	1		1	
Irregular + no drug	3.11(2.08–4.55)	< 0.001	2.47(1.84–3.91)	< 0.001

**Fig. 4:**
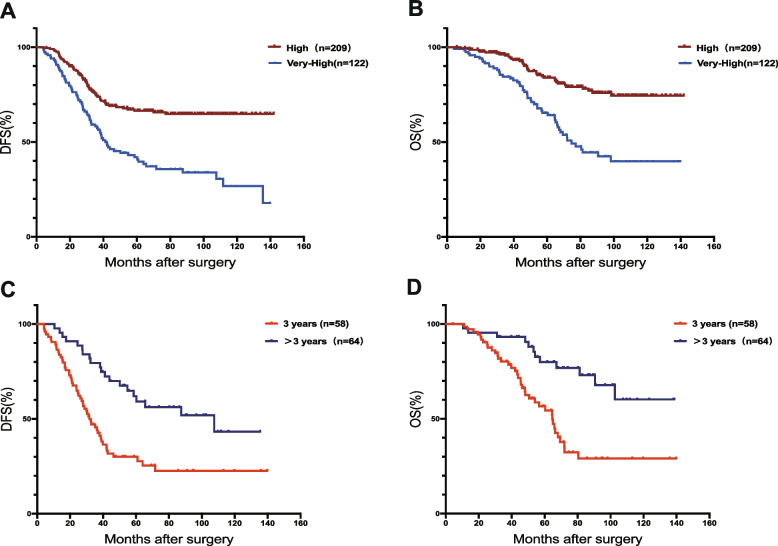
Prognostic analysis of 124 patients diagnosed with “very” high-risk: overall survival (**A**) and disease-free survival (**B**) between “very” high-risk patients and normal–high-risk patients; the overall survival (**C**) and disease-free survival (**D**) among different TKI maintenance treatment time. TKI: Tyrosine kinase inhibitor

Through above analysis, we found that the prognosis of patients with tumor diameter over 10 cm or mitotic rate over 10/50 HPF or extended radical resection was quite poor. We wondered whether such patients had a worse prognosis than other patients in high-risk patients, so we classified patients with more than 2 of the above three indicators as “very” high-risk patients, a total of 122 patients were finally selected. The “very” high-risk patients had significantly worse prognosis than normal high-risk patients (Fig. 4A, B). We divided them into two groups according to the length of TKI treatment: regular treatment (3 years) and continuous treatment (more than 3 years), the details shown in Table 3. There was no statistically significant difference between the two groups of patients in various clinical indicators, including gender, age, BMI, presence or absence of anemia, tumor location, tumor histological type, and tumor diameter. The prognosis of patients was improved, and the difference was statistically significant (DFS: *p* < 0.001, OS: *p* < 0.001, Fig. 4C, D).

**Table 3:** Univariate analysis of clinical factors in 122 patients with very-high risk GIST with different TKI treatment groups

Characteristic		3 years (*n* = 58)	Over 3 years(*n* = 64)	*P* value
Age	≤ 60	24	29	0.816
	> 60	34	35	
Sex	Male	36	40	0.714
	Female	22	24	
BMI(kg/m^2^)	≤ 22.78	29	34	0.413
	> 22.78	29	30	
Anemia	Yes	11	14	0.398
	No	47	50	
Tumor site	Stomach	11	13	0.217
	DU + SM	34	37	
	Others	13	14	
Histological variant	Spindle	36	39	0.091
	Epithelioid	17	16	
	Mixed	5	9	
Ki-67 index(%)	≤ 10	11	13	0.661
	> 10	47	51	

Based on the results of the above univariate prognostic analysis, we included the above statistically significant clinical factors into a multivariate analysis (Table 4) to evaluate the factors associated with the prognosis of patients with intermediate and high risk. The results are as follows: (1) among intermediate risk, patients with the impact of Independent factors associated with DFS included tumor location (*P* < 0.001) and KI-67 index (*P* = 0.008), but only postoperative TKI treatment was associated with postoperative overall survival (*P* < 0.001), and irregular targeted therapy of intermediate-risk patients was associated with poorer prognosis (HR 1.95, 95% CI 1.31–2.49, *P* < 0.001). For high-risk patients, the factors associated with both DFS and OS were tumor location, tumor diameter, Ki-67 index, and TKI treatment methods, and all P were less than 0.001, all of which were independent risk factors affecting prognosis; but except for In addition to the above factors, surgical method was associated with postoperative DFS (*P* < 0.001), while mitotic figures were associated with postoperative OS (*P* < 0.001). We found that the tumor was located in non-gastric patients (especially patients with non-gastroduodenal small intestine, HR 1.55, 95%CI 1.19–2.00, *P* < 0.001) tumor diameter > 10 cm (HR 2.63, 95%CI 2.09) − 4.03, *P* < 0.001), high-risk patients with mitotic figures > 10/50 (HR 2.74, 95%CI 2.00–3.76, *P* < 0.001) had a significantly worse overall prognosis than other patients. For “very” high-risk patients, prolonged postoperative TKI therapy can significantly improve the survival of patients (HR 0.43, 95% CI 0.15–0.66, *P* < 0.001).

**Table 4:** Multivariate survival analysis of intermediate- and high-risk patients

Variable	DFS		OS	
	HR (95%CI) *	*P* value	HR (95%CI) *	*P* value
Middle risk(*N* = 204)				
Primary tumor site				
Others vs. stomach	1.77(1.41–2.18)	< 0.001	–	–
Ki-67 index				
> 5 vs. ≤ 5	1.22(1.11–1.39)	0.008		
TKI therapy				
Irregular vs. regular	–	–	1.95(1.31–2.49)	< 0.001
High risk(*N* = 331)				
Primary tumor site		< 0.001		< 0.001
DU + SM vs. stomach	1.43(1.18–1.67)	0.008	1.33(1.07–1.59)	< 0.001
Others vs. stomach	1.79(1.28–2.19)	< 0.001	1.55(1.19–2.00)	< 0.001
Tumor size (cm)		< 0.001		< 0.001
5–10 vs. ≤ 5	1.12(1.04–1.31)	0.023	1.02(1.01–1.21)	0.037
> 10 vs. ≤ 5	3.22(2.39–5.48)	< 0.001	2.63(2.09–4.03)	< 0.001
Ki-67 Index(%)		< 0.001		< 0.001
5–10 vs. ≤ 5	1.11(1.09–1.34)	0.018	1.38(0.94–2.06)	0.061
> 10 vs. ≤ 5	2.49(1.89–3.27)	< 0.001	2.74(2.00–3.76)	< 0.001
Mitotic rate				
> 10/50 vs. ≤ 10/50	1.33(0.59–2.44)	0.231	1.88(1.31–2.76)	< 0.001
Surgery		< 0.001		0.331
Local excision vs. contained DTE	1.14(1.03–1.27)	0.019	1.55(0.71–3.16)	0.514
Local excision vs. extensive	2.97(2.01–4.09)	< 0.001	1.99(0.97–2.76)	0.089
TKI therapy				
Irregular vs. regular	2.71(2.09–3.29)	< 0.001	1.89(1.45–3.12)	< 0.001
Very high risk(*N* = 122)				
TKI therapy				
Over 3 years vs. 3 years	0.49(0.31–1.02)	0.061	0.43(0.15–0.66)	< 0.001

## Discussion

In our single-center, large-sample retrospective study, we explored the effect of treatment modalities at different risk levels on patient outcomes. Patients with different risk levels had different prognoses related to different clinical factors. For low-risk patients, surgery could achieve radical therapy. Among intermediate-risk patients, tumor location, Ki-67 index was related to DFS and OS. While for high-risk patients, those with non-gastric tumors, the higher tumor diameter, the higher mitotic rate, the overall prognosis was obviously worse than that of other patients. For some high-risk patients, prolonged postoperative imatinib therapy could significantly improve the survival of patients.

For the surgical treatment of GISTs, R0 resection is the current goal of radical surgery, but great controversy has emerged over the choice of surgical method, especially for endoscopic resection. Therefore, the 2020 edition of Gastrointestinal Stromal Tumor Diagnosis and Treatment Guidelines [21] point out that for those who cannot tolerate or refuse surgical resection, or for special sites or diameters < 2 cm, an experienced center should be selected for endoscopic resection. However, with the further improvement of medical technology, the technology of laparoscopic and robotic surgery has gradually improved. Many single-center and multi-center studies [9, 10, 22] have shown that in the surgical treatment of gastrointestinal tumors, laparoscopic and robotic surgery are not effective. Moreover, the safety and feasibility of such surgeries are uncertain. In our study, low-risk patients underwent tumor resection with different methods, including patients who underwent endoscopic resection (ESR group), received minimally invasive surgery group (MIS), accepted the traditional open surgery group. Take the safety and effectiveness into consideration, the less invasive surgery should be accepted in low-risk GIST patients.

The current criteria for assessing the risk of recurrence are mainly based on tumor location, tumor diameter, and mitotic figures, and are the criteria for assessing the need for targeted therapy after surgery [23, 24]. In our study, we found that tumor location, tumor size, surgical method, mitotic image, postoperative risk, and imatinib treatment were significantly associated with prognosis. The above factors were independent factors affecting the survival time (PFS, OS) of patients after surgery, and these results were basically consistent with those of previous studies [5, 25]. In addition, we found a direct association between TKI treatment modality and prognosis; therefore, we performed a separate survival prognostic analysis in 535 intermediate- and high-risk patients. We found that only the Ki-67 index was associated with postoperative DFS time and postoperative survival time. Interestingly, the presence or absence of TKI-targeted therapy did not affect patient outcomes. However, many previous studies have suggested [25–27] that the prognosis of intermediate-risk patients is basically consistent with the relevant clinical indicators of the risk assessment system. However, for the Ki-67 index, recent studies [28, 29] have recognized it as a prognostic factor. In sum, when judging the prognosis of patients with intermediate risk, in addition to paying attention to conventional indicators, we also need to pay attention to the treatment of Ki-67 and TKI. For intermediate-risk patients, except for tumor location, tumor size, and mitotic rate, we also should attach importance to the Ki-67 index.

As for high-risk patients, the exist guidelines is that the patients who at high risk of recurrence be recommended to receive least 3 years of adjuvant TKI treatment. But a phase II study, named as PESIST-5 [30], showed some patients who diagnosed with intermediate to high risk GIST could benefit from 5-year adjuvant imatinib, but 49% patients of the whole group in the study stopped the treatment before the end time. In our study, we raised “very” high risk, contained tumor diameters > 10 cm, Ki-67 index > 10%, and underwent extended radical resection, the prognosis of postoperative TKI maintenance therapy (more than 3 years) was significantly better than that of conventional treatment patients. Another study (SSG XXII, NCT 02413736) comparing 3- and 5-year adjuvant treatment is underway. João [31] and other scholars clearly pointed out in a review that preoperative neoadjuvant therapy can significantly reduce the tumor diameter and improve the radical cure rate for patients whose primary tumor is the stomach. However, there was no statistical difference between the high and low mitotic images of patients with PFS, and our findings were basically consistent with those of previous retrospective studies [18, 25]. In conclusion, in high-risk patients, preoperative evaluation becomes particularly important. Preoperative imatinib treatment can significantly improve the degree of radical surgery and improve the prognosis of patients with tumors greater than 10 cm in diameter. The maintenance time of TKI can be further extended when circumstances permit.

In sum, from our centers’ real-world study, we obtained the corresponding results by stratifying and segmenting 1015 patients who underwent surgical treatment. The value of surgical treatment of GIST is still unquestionable, but for intermediate- and high-risk patients, we need to pay attention to more than routine clinical factors including tumor location and diameter. The Ki-67 index, mitotic images, and the applicable population of TKI-targeted therapy after surgery also require attention. As our study is a retrospective research, it has certain limitations. Furthermore, multi-center prospective studies are required to verify the corresponding results.

## Supplementary Information

**Additional file file 1:** **Supplement file 1.**

## Data Availability

The datasets used and analyzed during the current study are available within the manuscript and its figures and tables.
