# Supplementary material for: Clinical characteristics and outcomes of gastrointestinal stromal tumor patients receiving surgery with or without TKI therapy: a retrospective real-world study
Source: World J Surg Oncol. 2023 Jan 23;21:21. doi: 10.1186/s12957-023-02897-y (PMC9869533; doi:10.1186/s12957-023-02897-y)
Supplement: Supplementary file 1 — Additional file file 1: Supplement file 1. [file 12957_2023_2897_MOESM1_ESM.zip › Supplement file-1.docx]

**Survival and clinical analysis of the whole group**

The last follow-up date of this study was December 31, 2021, and the median follow-up time was 76.95 months (23.53–145.91 months). As of the last follow-up date, 153 (15.1%) of the 1015 GIST patients in the whole group had tumor recurrence or progression, and 91 (8.97%) died of GIST recurrence or metastasis, 14 (1.37%) patients were lost to follow-up. For 153 patients with relapse or disease progression, we found that they mainly occurred in the peritoneum and/or liver, while a small number of patients also had metastases in the lung, bone, and lymph nodes. Through KM survival analysis, we found that the median DFS time and the distribution of the median overall survival time of the whole group of patients were 119.48 months and 133.47 months, respectively.

In univariate analysis, we found that tumor location, tumor size, surgical method, mitotic image, postoperative risk, and TKI treatment were significantly associated with prognosis (sup-Table-1). We then incorporated the above clinical factors into a multivariate analysis (COX regression): 1) Tumor location in the small intestine + duodenum (HR 2.15, 95% CI: 1.18–3.63, p=0.011), and other locations (HR 2.72, 95%) CI: 1.39–4.13, p=0.003); 2) Tumor diameter >5 cm [5–10 cm (HR 1.73, 95% CI: 1.29–2.33, p=0.009), >10 cm (HR 3.08, 95% CI: 1.44–5.15, p =0.001)]; 3) mitotic figures >5/50 [5–10/50 (HR 1.19, 95% CI: 1.01–1.72, p=0.021), >10/50 (HR 1.27, 95% CI: 1.03–2.18, p=0.009)]; 4) Ki-67 index >5% [5–10% (HR 1.14, 95% CI: 1.05–1.19, p=0.034), >10% (HR 3.79, 95% CI: 2.29–5.38, p=0.002)]. The above factors were independent influencing factors of postoperative recurrence. For different risk degree, the prognosis of high-risk patients is significantly worse than that of low- and intermediate-risk patients(sup-Fig-1A,B). In terms of tumor location, the progression-free survival (PFS) and OS of patients with a primary tumor in the stomach were significantly better than those of patients with tumors in other sites (p < 0.001 and p = 0.0232, sup-Fig-1C,D). Patients with a tumor diameter greater than 5 cm had a significantly worse prognosis than those with a tumor diameter less than 5 cm, and the PFS and OS had a statistical difference (p<0.0001, p=0.001, up-Figure 1E, F). Patients whose tumor mitotic rate less 5/50 HPF had a significantly well prognosis than those with over 5/50 HPF, especially in those over 10/50 HPF, and the PFS and OS had a statistical difference (p<0.0001, p<0.001, sup-Fig-1G, H). In addition, the Ki-67 index was also closely related to the prognosis; we found that the prognosis of patients with less than 5% on the Ki-67 index was significantly better than that of patients with more than 5%, and was particularly better than that of patients with more than 10%. Both PFS and OS had statistical differences, and p < 0.001 sup-Fig-1I, J).

**Analysis of clinical features and related factors among low-risk patients with different surgical methods**

According to postoperative pathology and risk assessment, there were 480 patients been considered as low-risk in the whole group; we divided 480 patients into three group depend on different surgical methods: endoscopic resection group (ESR, n=53) ,minimally invasive surgery group (MIS, n=274) ,the traditional open surgery group (Open, n=153). Among the three groups, we found differences in the distribution of BMI index, tumor location and tumor diameter: patients in the ESR and MIS group mainly happened in those whose tumor located in the stomach and have the smaller diameter, the smaller age, while the patients in the Open group were more likely to have higher BMI, tumor located in the non-stomach, and larger tumor diameters of patients were higher BMI index, higher proportion of tumor located in non-stomach, and larger tumor diameter (sup-Table 2).

In order to investigate the safety of different surgery methods, we taken the related clinical factors into consideration( sup-Table 3). The features of the three groups are as follows: 1) ESR group: less intraoperative blood loss, shorter operation time, shorter postoperative eating time but higher intraoperative replacement of surgical methods; 2) MIS group: shorter postoperative recovery time, shorter postoperative hospital stay, but longer operation time; 3) Open group: longer operation time, higher incidence of postoperative complications, and longer postoperative hospitalization time .
