# Supplementary material for: Clinical characteristics and outcomes of gastrointestinal stromal tumor patients receiving surgery with or without TKI therapy: a retrospective real-world study
Source: World J Surg Oncol. 2023 Jan 23;21:21. doi: 10.1186/s12957-023-02897-y (PMC9869533; doi:10.1186/s12957-023-02897-y)
Supplement: Supplementary file 1 — Additional file file 1: Supplement file 1. [file 12957_2023_2897_MOESM1_ESM.zip › Sup-Table1-3.docx]

Sup-Table 1：Univariate and multivariate survival (OS) analysis of clinicopathological characteristics of 1015 patients with gastrointestinal stromal tumor in the whole group

| **Variable** | **Univariate Analysis** | | | | **Multivariate Analysis** | |  |
| --- | --- | --- | --- | --- | --- | --- | --- |
|  | Object variable | Reference | HR（95％CI） | *P*-value | HR（95％CI） | *P*-value | |
| **Age** | ≥60 | <60 | 1.11(0.11-1.08) | 0.227 | 1.01(0.98-1.04) | 0.245 | |
| **Sex** | M | F | 0.84(0.49-1.44) | 0.259 | 1.27(0.73-2.18) | 0.396 | |
| **BMI(kg/m^2^)** | ≥22.78 | <22.78 | 0.89(0.51-1.56) | 0.403 | 0.89(0.51-1.57) | 0.692 | |
| **Anemia** | Yes | No | 0.98(0.87-1.98) | 0.421 | 0.88(0.50-1.55) | 0.651 | |
| **Primary tumor site** | DU+SM | Stomach | 3.35(1.38-8.12) | <0.001 | 2.15(1.18-3.63) | 0.011 | |
|  | Others |  | 3.29(2.13-5.10) | 0.008 | 2.72(1.39-4.13) | 0.003 | |
| **Primary tumor**  **Size（cm）** | 5-10 | <5 | 2.17(1.28-3.22) | 0.004 | 1.73(1.29-2.33) | 0.009 | |
|  | >10 |  | 3.41(1.32-6.00) | 0.001 | 3.08(1.44-5.15) | 0.001 | |
| **Mitotic Rate** | 5-10 | <5 | 1.15(1.03-1.76) | 0.018 | 1.09(1.01-1.72) | 0.021 | |
|  | >10 |  | 1.82(1.06-3.14) | 0.003 | 1.27(1.03-2.18) | 0.008 | |
| **Histological variant** | Epithelioid | Spindle | 1.51(0.26-1.98)  -2.12) | 0.463 | 1.01(0.78-1.98) | 0.290 | |
|  | Mixed |  | 1.41(0.94-2.12) | 0.095 | 1.02(0.11-1.81) | 0.187 | |
| **Ki-67 Index（%）** | 5-10 | <5 | 1.22(1.15-1.39) | 0.005 | 1.14(1.05-1.19) | 0.034 | |
|  | >10 |  | 3.10(1.13-6.17) | <0.001 | 3.79(2.29-5.38) | 0.002 | |
| **TKI therapy** | Irregular | Regular | 1.78(1.05-3.36) | 0.004 | 1.71(1.38-2.12) | 0.030 | |
|  | No-drug |  | 1.27(1.12-2.56) | 0.011 | 1.22(1.14-1.75) | 0.019 | |

Sup-Table 2：Comparison of clinical characteristics of different surgical methods in low-risk patients

| **Characteristic** | **N (%)** | **Group** | | | ***P*-value** |
| --- | --- | --- | --- | --- | --- |
|  |  | ESR(n=53) | MIS(n=274) | OPEN(n=153) |  |
| **Age** |  |  |  |  | 0.210 |
| **≤60** | 162(33.7) | 25(47.2) | 88(32.1) | 49(32.0) |  |
| **>60** | 328(66.3) | 28(52.8) | 186(67.9) | 104(68.0) |  |
| **Sex** |  |  |  |  | 0.067 |
| **Male** | 241(50.2) | 26(49.1) | 130(47.4) | 85(55.5) |  |
| **Female** | 239(49.8) | 27(50.9) | 144(52.)6 | 69(44.5) |  |
| **BMI(kg/m^2^)** |  |  |  |  | 0.011 |
| **≤22.78** | 329(68.5) | 23(43.4) | 206(75.2) | 100(65.3) |  |
| **>22.78** | 151(31.5) | 30(56.6) | 68(24.8) | 53(34.7) |  |
| **Anemia** |  |  |  |  | 0.371 |
| **Yes** | 41(9.3) | 6(11.3) | 24(8.8) | 11(7.2) |  |
| **No** | 439(90.7) | 47(88.7) | 250(91.2) | 142(92.8) |  |
| **Tumor site** |  |  |  |  | <0.001 |
| **Stomach** | 322(67.0) | 48(90.6) | 179(65.3) | 95(62.1) |  |
| **DU+SM** | 135(28.2) | 2(3.7) | 90(32.8) | 43(28.1) |  |
| **Others** | 23(4.8) | 3(5.7) | 5(1.9) | 15(9.8) |  |
| **Tumor Size（cm）** |  |  |  |  | <0.001 |
| **≤2** | 161(33.5) | 38(71.7) | 100(36.5) | 23(15.0) |  |
| **>2** | 329(66.5) | 15(28.3) | 174(63.5) | 130(85.0) |  |

Sup-Table 3：Analysis of surgical safety-related indicators in different groups of low-risk patients

| **Characteristic** | **Group** | | | **P-value** |
| --- | --- | --- | --- | --- |
|  | ESR(n=53) | MIS(n=274) | OPEN(n=153) |  |
| **Operation time (Mean ± SD，mins )** | 118**±**33.1 | 135**±**38.5 | 146**±**32.1 | 0.014 |
| **Bleeding volume (Median ,range)** | 29(2-65) | 38(10-100) | 96(15-210) | <0.001 |
| **Recovery feeding (Median ,range)** | 6.4**±**2.1 | 7.1**±**2.9 | 7.8**±**3.3 | 0.032 |
| **Operation complication** |  |  |  | 0.025 |
| **Yes** | 4(8.2) | 22(8.4) | 25(16.3) |  |
| **No** | 49(91.8) | 222(91.6) | 128(83.7) |  |
| **Convert to another method** |  |  |  | 0.011 |
| **Yes** | 7(13.2) | 13(4.7) | / |  |
| **No** | 46(86.8) | 261(95.3) | / |  |
| **Second-operation(n,％)** | 1(1.9) | 6(2.2) | 5(3.3) | 0.078 |
| **Hospital stays(Mean ± SD, days）** | 9.1**±**3.7 | 8.7**±**3.5 | 11.1**±**4.6 | <0.001 |
