# Supplementary figures and images for: Clinical characteristics and outcomes of gastrointestinal stromal tumor patients receiving surgery with or without TKI therapy: a retrospective real-world study
Source: World J Surg Oncol. 2023 Jan 23;21:21. doi: 10.1186/s12957-023-02897-y (PMC9869533; doi:10.1186/s12957-023-02897-y)

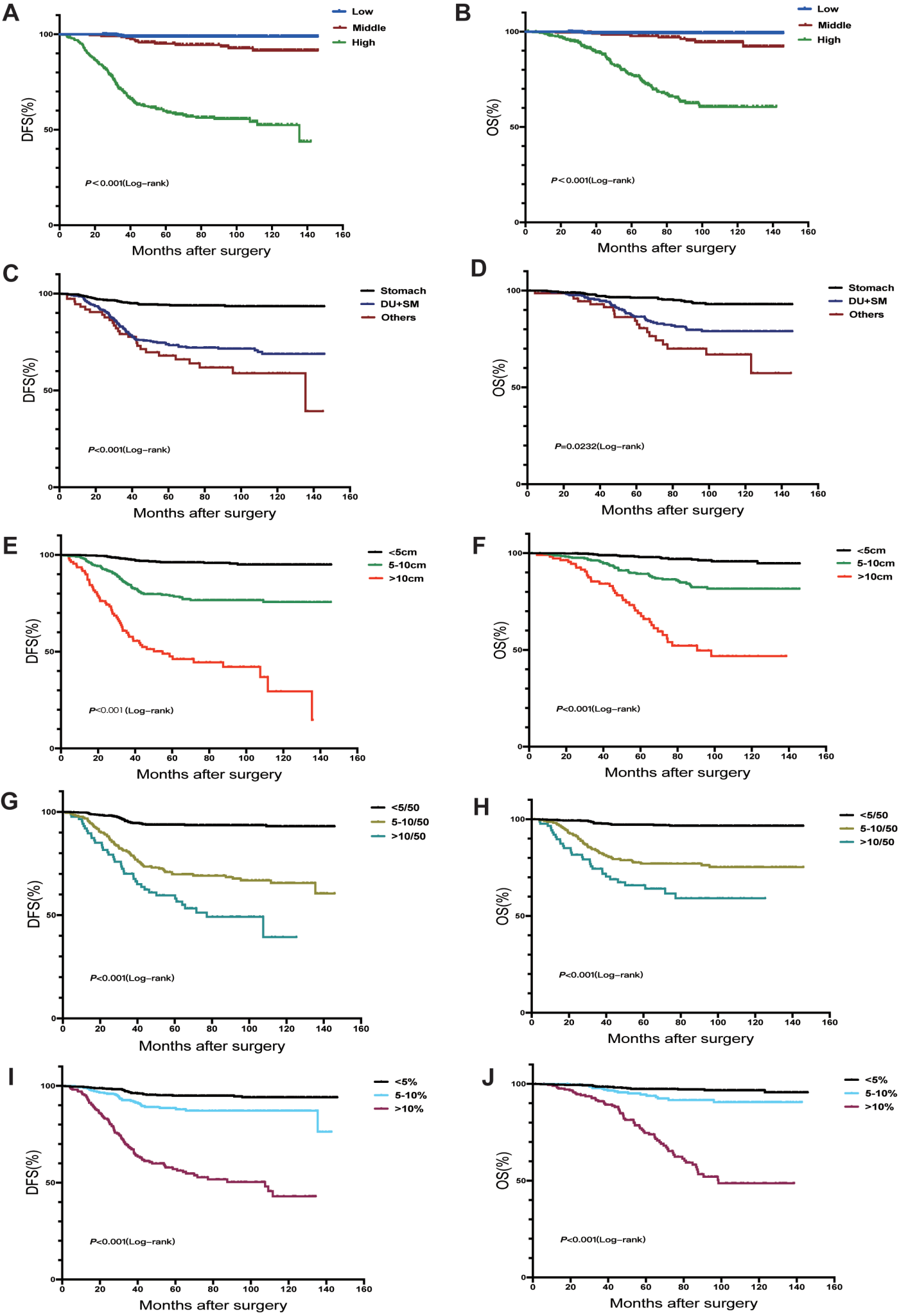

Supplement: Supplementary file 1 — Additional file file 1: Supplement file 1. [file 12957_2023_2897_MOESM1_ESM.zip › sup-Figure 1.pdf]
